# Rare Postoperative Complication of Esophageal Atresia after Open Thoracotomy Repair in Newborn—Lung Torsion: A Case Report

**DOI:** 10.3390/pediatric16030060

**Published:** 2024-08-15

**Authors:** Szymon Gryboś, Viera Karaffová, Katarina Klapačová

**Affiliations:** 1 Department of Pediatric Surgery, Childrens Faculty Hospital in Košice, Tr. SNP 1, 04011 Košice, Slovakia; szymon.jakub.grybos@dfnkosice.sk (S.G.); katarina.klapacova@dfnkosice.sk (K.K.); 2 Department of Morphological Disciplines, University of Veterinary Medicine and Pharmacy in Košice, Komenského 73, 04181 Košice, Slovakia

**Keywords:** esophageal atresia, lung torsion, postoperative complication, newborn

## Abstract

In this case report, we described a rare complication—lung torsion after esophageal atresia repair in a newborn. Torsion of the lung is a critical condition in which either the entire lung or a lung lobe twists, leading to occlusion of blood vessels and airways. The patient’s clinical condition was poor after the primary operation. An emergency thoracotomy showed 180° torsion of the right upper lobe (RUL) and right middle lobe (RML). After detorsion, perioperatively, the lung was gradually reperfused and had a normal appearance. After surgery, the patient was unstable, which culminated in a fatal end (bradycardia, reperfusion injury). Immediate intervention can preserve the affected lung or lung lobe. However, pulmonary torsion typically has a poor prognosis due to misdiagnosis and delayed treatment. Additionally, diagnosis in the neonatal period is even more challenging because the clinical symptoms are nonspecific. In any case, the question is whether detorsion is the right solution or whether a segmentectomy is necessary.

## 1. Introduction

Esophageal atresia (EA) is a congenital developmental defect with a documented prevalence ranging from 1:3000 to 1:10,000, requiring urgent surgical intervention. The disorder is characterized by the failure of a segment of the esophagus to develop—the esophagus lacks continuity, and one or both ends (proximal or distal) end in a blind pouch. The most common early complications include anastomotic leakage, recurrent tracheoesophageal fistula, and anastomotic stricture [1].

Lung torsion is a rare condition that can present after thoracic cavity disruption. Common causes of lung torsion include thoracotomy, lung transplantation, and trauma. The actual incidence of this complication is unknown in children. However, spontaneous cases have also been reported [2]. Torsion of the lungs itself is challenging in children, especially in newborns, due to their age, as we can only rely on subtle clinical signs.

In the previously published pediatric literature, we have noted three papers regarding complications of esophageal atresia repair, such as lung torsion in newborns (Table 1). Although the clinical presentation of the described case studies was comparable, the management of each case was different. In two out of the three studies, a late segmentectomy had to be performed, despite the primary detorsion.

## 2. Case Presentation

### 2.1. Clinical History

**Figure 1:**
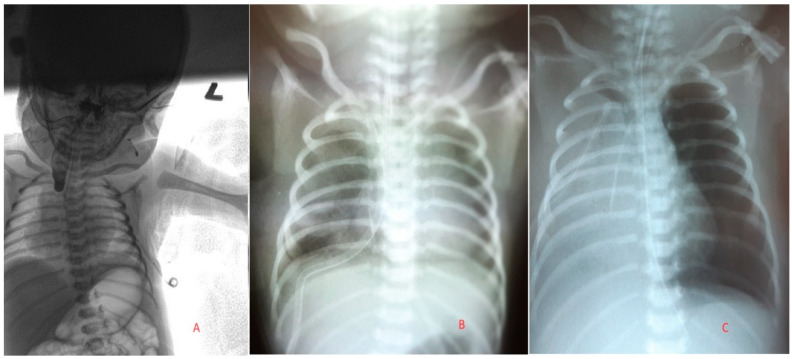
(**A**)—X-ray-showing the upper end of the esophagus at the level of T4-5, dilated intestinal loops filled with air. (**B**)—X-ray image taken 2 h post-operation. (**C**)—X-ray taken 6 days after surgery and after re-drainage of the chest.

A full-term eutrophic newborn from a second high-risk pregnancy was born spontaneously at 38 weeks of gestation with a birth weight of 3370 g. Immediate postnatal adaptation was challenging, with the presence of dyspnea, grunting, desaturation, subcyanosis, and an Apgar score of 8/7. Initial examination revealed esophageal atresia during probing. However, other routine postnatal screening examinations were normal. Due to developing signs of respiratory distress, the child was intubated and mechanically ventilated. Hypoglycemia was corrected with an infusion. An X-ray with contrast confirmed a congenital gastrointestinal anomaly—esophageal atresia. Parenteral nutrition, antibiotic therapy (Ampicillin + Gentamicin), and intensive monitoring of the patient were prescribed. A chest X-ray, performed immediately after birth following the administration of 1 mL of oral contrast, confirmed the presence of a congenital defect—esophageal atresia with a distal tracheoesophageal fistula (Figure 1A).

The surgeon assessed the condition as EA with TEF Vogt IIIB and recommended surgical intervention.

### 2.2. Surgical Intervention

After prior preparation, the child was operated on the third day of life under general anesthesia. A right-sided thoracotomy with a transpleural approach was performed, with no perioperative complications. The surgeon can choose a transpleural or extrapleural approach. However, the extrapleural approach is more difficult to achieve because, in newborns, the pleura is very fragile and can be damaged. This happened in our case as well, where the surgeon planned for an extrapleural approach but had to continue with a transpleural procedure due to the poor quality and damage to the pleura. During the operation, an end-to-end anastomosis was performed, the tracheoesophageal fistula was closed, a chest drain was placed, and two central venous catheters were inserted into the left subclavian vein and the left femoral artery. Before closure, the lungs were ventilated with controlled ventilation, which continued until all collapsed lung areas were fully expanded. Postoperatively, the child was stable, continuously analgosedated via a paravertebral catheter (Sufenta + Marcain), and on mechanical ventilation. Control chest X-rays showed atelectasis in the upper and middle lobes of the right lung (Figure 1B). However, biochemical analysis revealed elevated inflammatory parameters (C-reactive protein (CRP), total leukocytes (WBC), and interleukin-6 (IL-6)), in presumed association with lung inflammation and surgical performance.

On the second postoperative day, the child’s ventilation status worsened, requiring an increase in the ventilation settings and an oxygen requirement of 40–50%. The X-ray showed homogeneous shadowing of the right lung field. Given the presence of a chest drain that was draining neither air nor fluid, re-drainage was performed. Laboratory parameters showed an increase in inflammatory activity, and the antibiotic therapy was changed to Vancomycin and Ceftazidime. The attending physicians estimated a severe lung infection. Despite repositioning the drain, there was no improvement in the child’s condition. The drain extracted a small amount of sanguineous content after being connected to active suction (Figure 1C).

**Figure 2:**
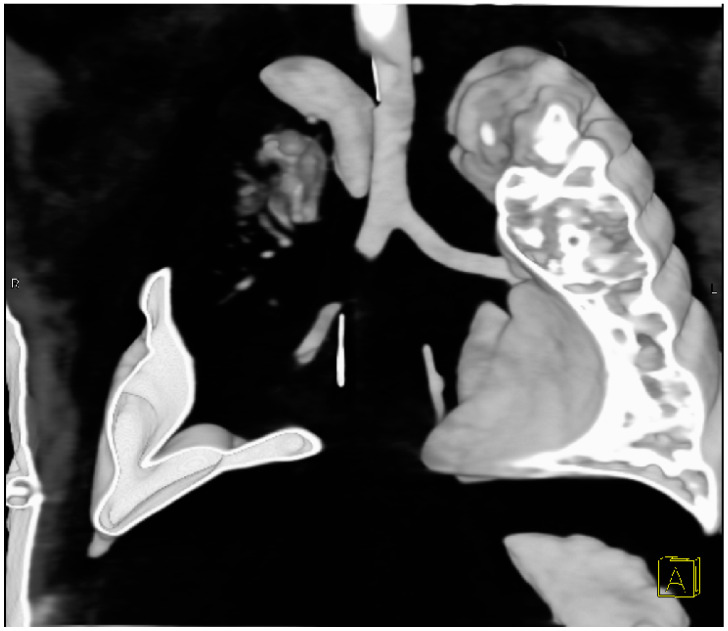
CT bronchography—occlusion of the right main bronchus 6 mm distal to the bifurcation, extending 8 mm.

The child’s ventilation continued to worsen, with the entire right lung field non-aerated. Given this, a suspected obstruction of the right bronchus was considered. On the 7th postoperative day, bronchoscopy was performed, revealing a narrowing of the right main bronchus immediately after its origin, with unclear etiology. Consequently, a Computer Tomography scan of the chest was indicated. Virtual CT bronchoscopy showed an occlusion of the right main bronchus 6 mm distal to the bifurcation, extending for 8 mm. Caudally, only a short residual part of the medial lobar bronchus, ending blindly, was observed. The origin of the right superior lobar bronchus was not visible, with only its residual blindly ending branches registered (Figure 2).

**Figure 3:**
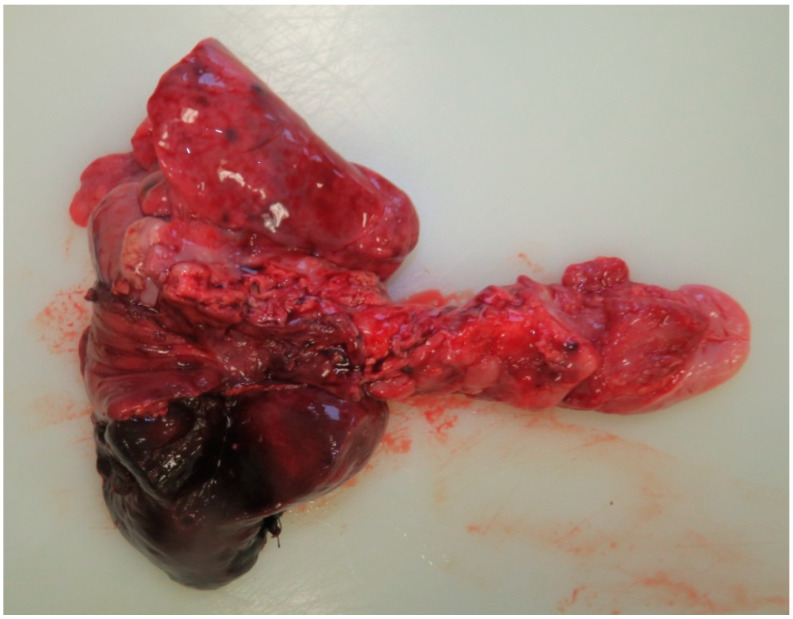
Autopsy finding.

The child remained significantly unstable, requiring 100% oxygen, with worsening signs of respiratory insufficiency. Transiently, the child was connected to high-frequency ventilation, which was not tolerated, so the mode was changed back to conventional ventilation with high parameters. Despite the deteriorating and critical condition, which required catecholamine support (Dobutamin + Dopamin), surgical revision was indicated on the 7th postoperative day. During the revision, infarction of the upper and middle lobes of the right lung was found. A 180-degree rotation of the right middle lobe in a clockwise direction was identified, causing traction and compression of the vessels leading to the upper lobe of the lungs. The anastomosis itself and the closed TEF were not compromised. The perioperative course was complicated by hypotension and bradycardia, with the child remaining unstable, requiring high ventilatory support, FiO_2_-1.0, and continuously administered adrenaline during the operation. Due to the patient’s clinical condition, intraoperative detorsion was performed, after which the lungs were ventilated, gradually appearing vital. After transfer from the operating room to the ward, the child became bradycardic and desaturated during transport. CPR with chest compressions was initiated. Upon arrival at the ward, CPR was expanded, and a bolus of adrenaline was administered without response. Asystole was present, and lethal exitus was confirmed. The cause of death, according to the autopsy report, was respiratory failure due to torsion and hemorrhagic infarction of the upper and middle lobes of the right lung, concomitant with acute interstitial pneumonia of the left lung with respiratory failure (Figure 3).

**Figure 4:**
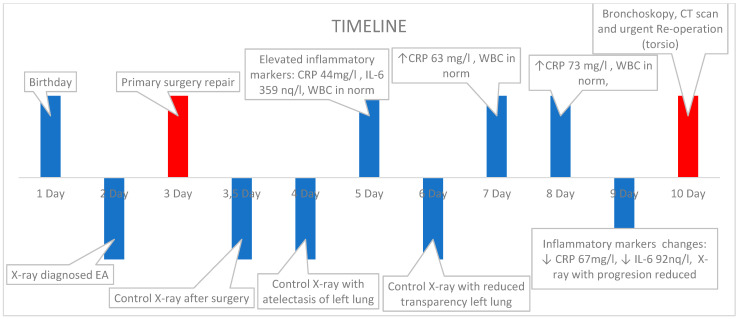
Timeline of case.

The entire timeline of this case is summarized in Figure 4.

## 3. Discussion

Esophageal atresia with tracheoesophageal fistula (TEF) is a rare congenital defect affecting the trachea and esophagus. Despite its potential to cause serious health problems, outcomes have improved significantly in recent years. This improvement is due to several factors, including advancements in surgical methods, specialized anesthetic care, and specific ventilator support. These developments have increased survival rates, even for premature infants with very low birth weights [6].

The most common procedure used to rectify the EA with TEF is a right thoracotomy, which encompasses the separation of the abnormal communication between the trachea and the esophagus and a subsequent primary end-to-end anastomosis of the esophagus. Nevertheless, postoperative complications such as anastomotic leakage remain a major challenge for surgeons, due to their occurrence, despite the advents in modern surgical care. The most common complications include anastomosis leakage, tension pneumothorax, and sepsis [7].

The pathophysiology is ambiguous. The literature attributes physical findings of lobe torsion to fever, tachycardia, and loss of breath sounds over the affected lung field. Radiological findings are more specific, such as rapid opacification or a change in the position of the affected lobe on a series of X-rays. Bronchoscopy and CT scans play a significant role in early diagnosis. Bronchial occlusion observed during bronchoscopy suggests a possible diagnosis and differentiates it from bronchial obstruction. Early diagnosis and surgical treatment are crucial-detorsion, or resection if the lobe is not viable. The decision between resection and detorsion alone of the lung lobe with doubtful viability is controversial. Some authors recommend resecting the affected lobe to prevent possible embolism [8].

The differential diagnosis of lung torsion includes several possibilities like hemorrhage, pneumonia, leakage of anastomosis, and aspiration [9].

An argument against detorsion is primarily the risk that necrotic tissue and inflammatory mediators may escape into the systemic circulation, potentially triggering a systemic inflammatory response leading to multiorgan failure and death, seen in our case, and we agree with that view [10].

Earlier reoperation would likely be beneficial, but since this complication is extremely rare in the pediatric population, the diagnostic process may take longer. Our timing is similar to those found in the literature. Another challenge is the extrapleural approach to thoracotomy, which would likely prevent torsion necrosis, but this is not always feasible due to anatomical conditions and the quality of the pleura in the neonatal period. Certain new modern surgical techniques, such as thoracoscopic surgical solutions, are beneficial in reducing the number of complications.

Similarly, Koziarkiewicz et al. [5] pointed out that the persistent atelectasis of a lung/lobe after thoracotomy should call attention to the possibility of torsion. We have also noticed this state of affairs in our case.

Yang et al. [4] elucidated the role of tissue elasticity, pleural adhesions, and vascular dynamics in predisposing individuals to lung torsion, but we are of the opinion that it involves multiple factors: the performance and type of anesthesia, inflammation, and surgical trauma to the lungs [10].

Moreover, to the best of our knowledge, the most comprehensive review in this field so far was carried out by the authors of Yang and Oliver et al. [3,4] in their studies, wherein they described nine cases of lung torsion in the pediatric population, including mostly after burns and abdominal trauma, spontaneously in bronchitis, the occlusion of the ductus arteriosus, a thoracotomy for hiatal closure, but also a case after esophageal atresia.

Collectively, the seminal contributions of Dr. Oliveiro, Dr. Eun Mi Yang, and Dr. Koziarkiewicz have propelled our understanding of lung torsion to new heights, as this is a very rare complication that a pediatric surgeon may encounter once in a lifetime or never. This discussion highlights the significant impact of each author’s research on advancing our understanding of lung torsion and improving patient care.

We agree with the latest findings on the issue from Dr. Jalota. She reports that the lung cannot go through detorsion, or, if detorsion fails, then a lobectomy must occur. Detorsion must be performed within the first few hours of diagnosis to save a viable lung. If the wait is any longer, the lung may already have irreversible ischemic damage, where it may be safer to perform a resection without detorsion. This would prevent the inflammatory markers that build up during the torsion from leaking out into the rest of the body and causing multiorgan failure [11].

## 4. Conclusions

Bronchoscopy and CT scans play vital roles in confirming the diagnosis but increased attention must be paid to atelectasis after surgery. The decision between detorsion and resection hinges on factors such as tissue viability and the risk of systemic complications.

For damaged tissue, it is advisable to maintain the lung in its rotated position until the pulmonary veins have been clamped to prevent the release of inflammatory markers into the systemic circulation. Once clamped, a clinical decision can be made regarding whether to proceed with detorsion of the lung or to perform a resection. According to previous studies, however, delayed segmentectomy was indicated in several cases; therefore, we suggest that it should be recognized as a lege artis procedure in the primary treatment. Detorsion can be performed only within the first few hours of diagnosis to save a viable lung, but it is very difficult in newborns.

These research studies have shown the importance of making the right decisions during surgery. They indicate that even more radical measures, such as resecting infarcted lungs rather than removing them, can be life-saving. This helps to control the gradual release of inflammatory markers and necrotic tissue, reducing the risk of a sudden surge that could result in fatal multiorgan failure.

## Figures and Tables

**Table 1:** Summary of literature about lung torsion in EA. Refs. [3,4,5] Legend: RML—right middle lobectomy.

Authors	Year	Approach and Type of Execution	Reoperation Time after Primary Surgery
Oliveira et al. [3]	2007	Transpleural, detorsion 90°	7 days
Yang et al. [4]	2013	Transpleural, detorsion 180°	3 days
		Segmentectomy RML	10 days
Koziarkiewicz et al. [5]	2014	Transpleural, detorsion 180°	8 days
		Segmentectomy RML	5 months

## Data Availability

The datasets used and/or analyzed during the current study are available from the corresponding author on reasonable request.
